# An obesity-associated gut microbiome reprograms the intestinal epigenome and leads to altered colonic gene expression

**DOI:** 10.1186/s13059-018-1389-1

**Published:** 2018-01-23

**Authors:** Yufeng Qin, John D. Roberts, Sara A. Grimm, Fred B. Lih, Leesa J. Deterding, Ruifang Li, Kaliopi Chrysovergis, Paul A. Wade

**Affiliations:** 1 Eukaryotic Transcriptional Regulation Group, Epigenetics and Stem Cell Biology Laboratory, National Institute of Environmental Health Sciences, Research Triangle Park, NC 27709 USA; 2 Integrative Bioinformatics Group, Epigenetics and Stem Cell Biology Laboratory, National Institute of Environmental Health Sciences, Research Triangle Park, NC 27709 USA; 3 Mass Spectrometry Research & Support Group, Epigenetics and Stem Cell Biology Laboratory, National Institute of Environmental Health Sciences, Research Triangle Park, NC 27709 USA

**Keywords:** Microbiome, Obesity, Cancer, Colorectal cancer, Epigenetics, Transcription factor, Gene expression, Histone methylation, Histone acetylation

## Abstract

**Background:**

The gut microbiome, a key constituent of the colonic environment, has been implicated as an important modulator of human health. The eukaryotic epigenome is postulated to respond to environmental stimuli through alterations in chromatin features and, ultimately, gene expression. How the host mediates epigenomic responses to gut microbiota is an emerging area of interest. Here, we profile the gut microbiome and chromatin characteristics in colon epithelium from mice fed either an obesogenic or control diet, followed by an analysis of the resultant changes in gene expression.

**Results:**

The obesogenic diet shapes the microbiome prior to the development of obesity, leading to altered bacterial metabolite production which predisposes the host to obesity. This microbiota–diet interaction leads to changes in histone modification at active enhancers that are enriched for binding sites for signal responsive transcription factors. These alterations of histone methylation and acetylation are associated with signaling pathways integral to the development of colon cancer. The transplantation of obesogenic diet-conditioned microbiota into germ free mice, combined with an obesogenic diet, recapitulates the features of the long-term diet regimen. The diet/microbiome-dependent changes are reflected in both the composition of the recipient animals’ microbiome as well as in the set of transcription factor motifs identified at diet-influenced enhancers.

**Conclusions:**

These findings suggest that the gut microbiome, under specific dietary exposures, stimulates a reprogramming of the enhancer landscape in the colon, with downstream effects on transcription factors. These chromatin changes may be associated with those seen during colon cancer development.

**Electronic supplementary material:**

The online version of this article (10.1186/s13059-018-1389-1) contains supplementary material, which is available to authorized users.

## Background

In eukaryotic cells, chromatin comprises a complex consisting of DNA, RNA, and proteins where covalent modifications correlate with regulation of gene expression [1]. Dietary factors have been shown to induce epigenome changes, including histone modifications [2, 3]. With the spread of the Western lifestyle, including consumption of an obesogenic diet high in fat, obesity rates have continued to rise worldwide during the last few decades [4]. Obesity is associated with low-grade chronic inflammation, which is a likely precipitating factor for numerous complications, including type 2 diabetes, cardiovascular disease, breast cancer, and colorectal cancer [4, 5]. Understanding how the host epigenome responds to diet and obesity may provide mechanistic insights into obesity-associated diseases.

The gut microbiota resides on the intestinal mucosal surfaces and plays an important role in food digestion, energy harvest, immune development, and epithelial homeostasis [6]. It induces both local effects in the gut, as well as alterations in distant organs through stimuli generated by bacteria, structural bacterial components, and microbial metabolites [7]. In particular, the microbiome can generate numerous bioactive compounds important to host physiology, including short chain fatty acids (SCFAs), the majority of which are acetate, propionate, and butyrate [8], choline metabolites, and lipids [6]. The SCFAs are rapidly adsorbed from the colonic lumen and constitute a preferred energy source for colonic epithelial cells. Dysbiosis of the gut microbiome has been shown to alter both the transcriptome and proteome of intestinal epithelial cells [9, 10] and numerous studies have identified associations between gut microbiome alterations and host diseases, including obesity, diabetes, cardiovascular disease, and colon cancer [8, 11–13].

Given the important roles of the gut microbiome in regulating host physiology and gene expression, a better understanding of the relationship between the host and the symbiont microbiota should provide new insights into human health and disease risk. Here, we employed a mouse model of diet-induced obesity to characterize molecular features of the interplay between host epigenome, gut microbiota, and diet. The murine gut microbiome and its metabolites were altered by an obesogenic diet in a manner that preceded the development of obesity. Diet-induced obesity led to altered acetylation of lysine 27 on histone H3 (H3K27ac) and to altered monomethylation of lysine 4 on histone H3 (H3K4me1) at numerous loci, with concomitant changes in the expression of genes that were functionally relevant to intestinal cancers. Transplantation of bacteria from obese, but not control, animals into germ-free mice was sufficient to recapitulate high fat-associated epigenetic changes, in a diet-dependent manner. Our study provides a better understanding of the complex interplay between diet, host gene expression, host epigenome, and the gut microbiome.

## Results

### Gut microbiome was shaped by diet prior to the development of obesity in a sex-specific manner

**Fig. 1:**
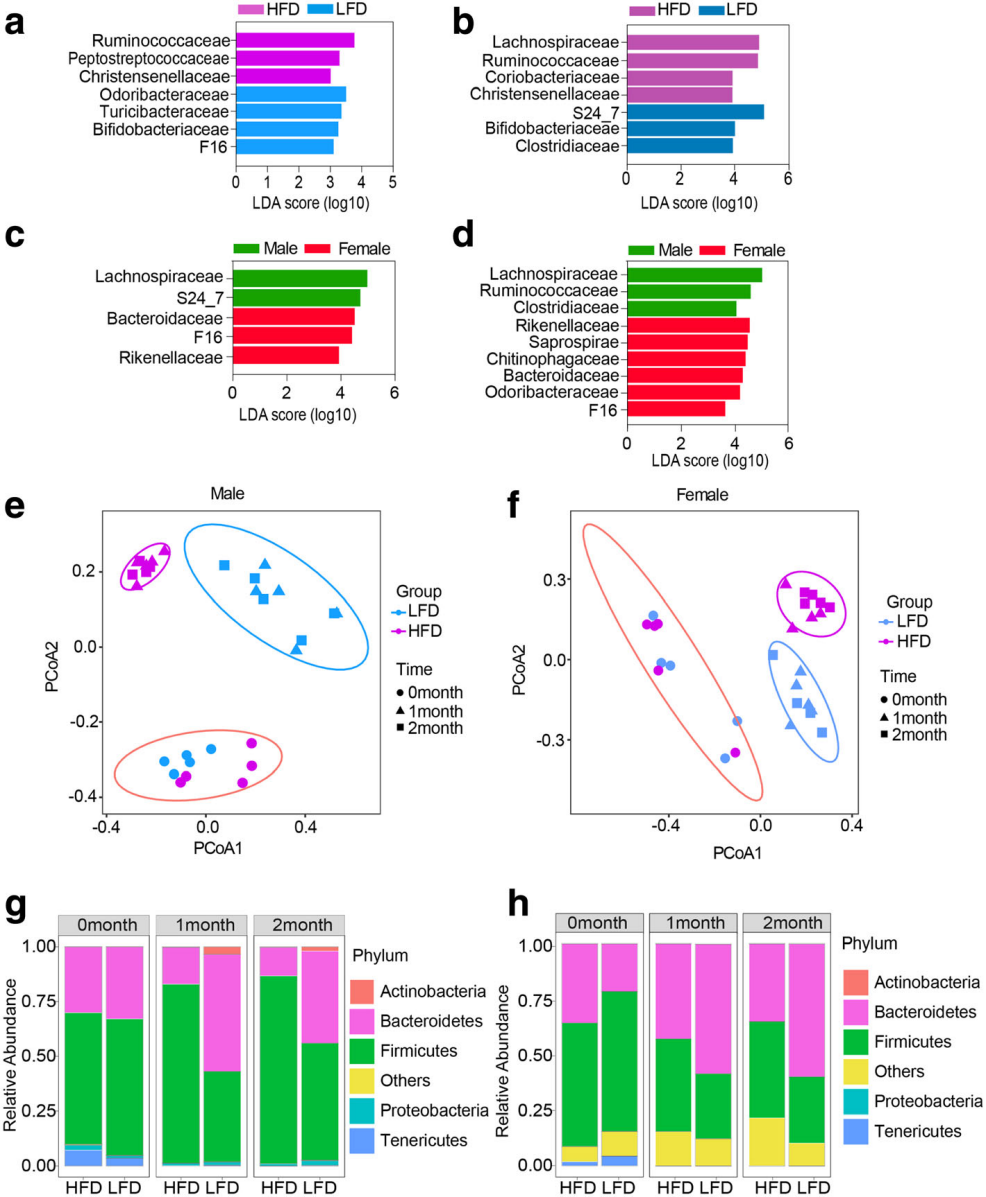
High fat diet shaped host microbiota prior to the appearance of obesity in a sex-dependent manner. **a**, **b** Linear discriminant analysis effect size (LDA) at family level for bacteria from male (**a**) and female (**b**) mice on different diets for 20 weeks. **c**, **d** LDA showed differentially enriched microbiota in HFD (**c**) and LFD (**d**) groups in male and female mice at family level. **e**, **f** Principal coordinate analysis (PCoA) of Bray-Curtis distance for bacteria at 0, 1, and 2 months of the two diets in male (**e**) and female (**f**) mice. **g**, **h** Relative abundance of bacteria at phylum level in male (**g**) and female (**h**) mice fed a HFD or LFD for 0, 1, and 2 months of the two diets. n = 10 per group for 16s sequencing analysis; n = 4–5 per group for the time-course study

To evaluate the contribution of diet and obesity to changes in host gut microbiota, male and female C57BL/6 mice were fed either a control, low fat diet (LFD), or high fat diet (HFD) for 20 weeks (Additional file 1: Table S1). The HFD caused a dramatic increase in body weight in both sexes (Additional file 2: Figure S1a). Consistent with other studies, we observed significant diet-dependent changes in microbial ecology in both males and females (Additional file 2: Figure S1b–f; Additional file 2: Figure S2a–e; Additional file 3: Table S2a, b). Through linear discriminant analysis (LDA) effect size (LEfSe) (LDA score > 2) [14], we found that families *Ruminococcaceae*, *Peptostreptococcaceae*, and *Christensenellaceae* were enriched in male obese mice, while the families *Odoribacteraceae*, *Turicibacteraceae*, *Bifidobacteriaceae*, and F16 were enriched in male lean mice (Fig. 1a). Comparing female obese mice and lean mice, we found families *Ruminococcaceae*, *Christensenellaceae*, *Lachnospiraceae*, and *Coriobacteriaceae* were also enriched in obese mice, while *Bifidobacteriaceae*, S24_7, and *Clostridiaceae* were enriched in lean mice (Fig. 1b).

Obesogenic diets, such as the HFD employed here, induce sex-specific patterns of adipogenesis [15], which involves a complex and highly orchestrated program of gene expression. Within 5 weeks, male mice on the HFD became moderately obese by gaining 22% more weight than mice on the LFD [16], while females took more than 10 weeks to reach this metric (Additional file 2: Figure S1a). In order to understand the relationships between sex-specific weight gain, diet, and the microbiome, we compared the gut microbiome composition between two genders. We found that the gut microbiome was significantly different between males and females independent of diet (Fig. 1 c, d; Additional file 2: Figure S3a–d). At the phylum level, the diet-dependent changes in relative abundance of the large taxa *Bacteriodetes* and *Firmicutes* differed by sex (Additional file 2: Figure S3c, d). Additionally, there were sex-specific differences in bacterial composition on both the HFD (Fig. 1c) and the LFD (Fig. 1d). Thus, the differential weight gain between males and females may reflect differences in composition and physiology of the respective gut microbiota.

To understand how diet and obesity change the gut microbiota, we sequenced the 16S rDNA from fecal samples collected from male and female mice before and after exposure to the HFD and LFD for 4 and 8 weeks (Additional file 3: Table S2c, d). PCoA showed that diet could shape the gut microbiota in as little as one month in both sexes (Fig. 1 e, f) as we observed discriminative separation between the two groups. The relative abundance of different bacteria began to resemble that of mice that were on the HFD for 20 weeks with high abundance of *Firmicutes* and low *Bacteroidetes* levels (Fig. 1 g, h), suggesting that, to some extent, gut microbiota composition reflects the host diet rather than obesity itself. Compared to males, the increase in levels of *Firmicutes* following administration of the obesogenic diet is substantially slower in females (Fig. 1 g, h). As *Firmicutes* are associated with increased energy harvest from food, this difference between the male and female microbiome may be causally associated with sex-dependent weight gain on an obesogenic diet.

Short chain fatty acids (SCFA) are a key metabolite produced by certain gut bacteria that provide an important source of energy for colonic epithelia [8]. In order to determine whether alterations in gut microbiota composition also changed the production of SCFA, we measured SCFA levels in fecal samples by GC-MS. We detected ten SCFA, the most prevalent being acetate, butyrate, and propionate. Interestingly, we found butyrate levels were significantly decreased in male mice fed HFD (48.0 ± 10.2 μg/g fecal sample) compared to LFD (143.7 ± 36.5 μg/g fecal sample) (Additional file 2: Figure S3e), while in female mice we observed more modest decreases in butyrate on the HFD (38.0 ± 14.2 μg/g fecal sample) compared to LFD (62.9 ± 14.4 μg/g fecal sample) (Additional file 2: Figure S3f). Overall, these findings suggested that there were both qualitative and quantitative differences in the gut microbiome and their metabolites between obese and lean mice and that the differences were sex specific.

### Diet and obesity modified the enhancer landscape and transcriptome in colon epithelium

**Fig. 2:**
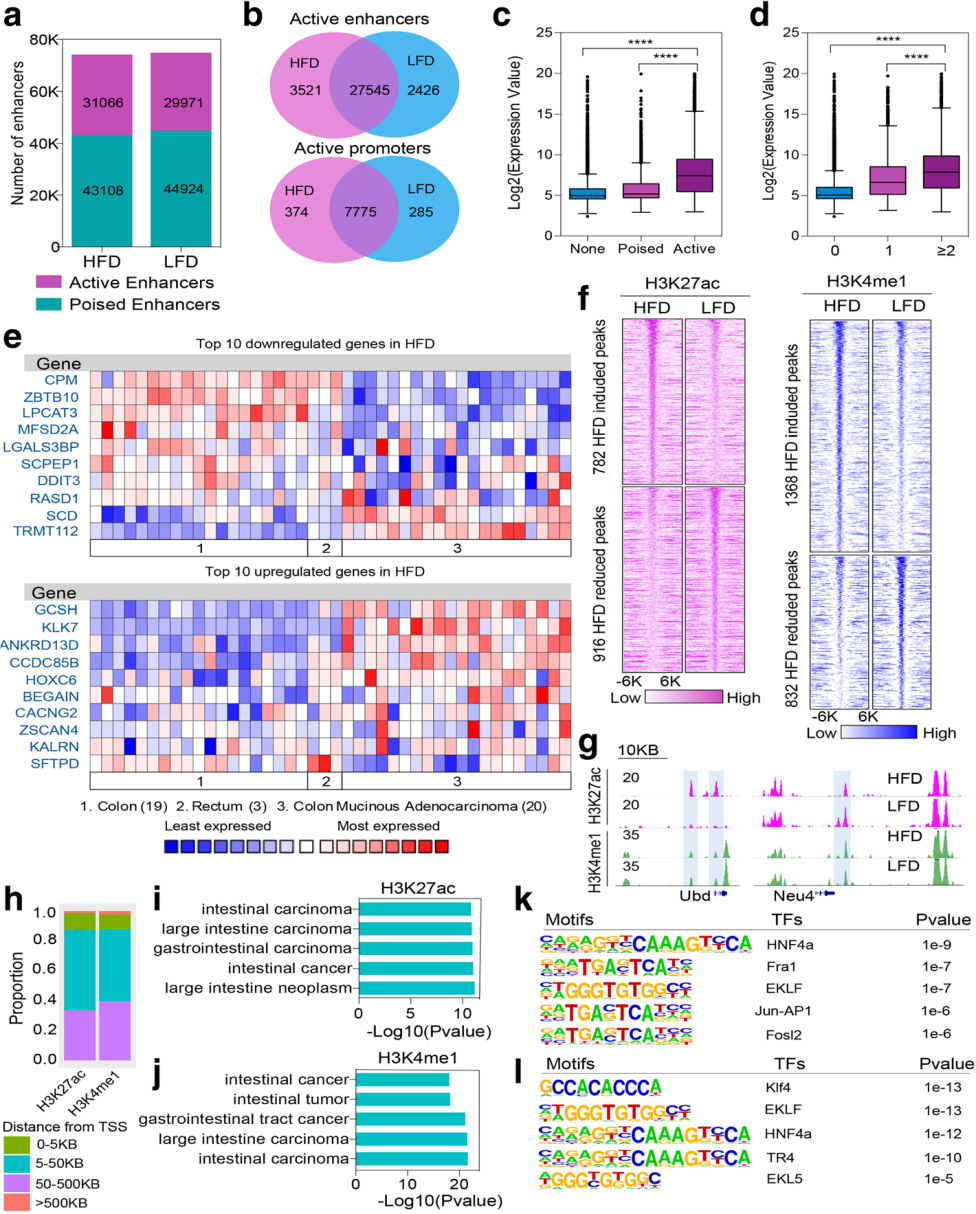
Diet and obesity altered the host transcriptome and epigenome. **a** Numbers of active (marked with both H3K27ac and H3K4me1) and poised enhancers (marked with H3K4me1 only) in obese and lean mice. **b** Overlap analysis of active enhancers and active promoters in obese and lean mice. **c** Expression levels at genes with none, poised, or active enhancers in colonic epithelium from animals on HFD. **d** Expression levels at genes with none, one, or more than one active enhancer in colonic epithelium in animals on LFD. **e** Oncomine analysis of differentially expressed genes from animals on HFD compared to differentially regulated genes from normal colon, normal rectum, and colon mucinous adenocarcinoma (groups 1, 2, and 3, respectively). **f** Heatmap of different enrichment loci of H3K27ac and H3K4me1 from colonic epithelium in mice on different diets. **g** Representative genome browser shot of differentially enriched loci of H3K27ac and H3K4me1 from colonic epithelium in mice on different diets. **h** Proportion of genes with different distances from differentially enriched loci for H3K27ac and H3K4me1 to transcription start site. **i**, **j** IPA analysis of differential enrichment loci of H3K27ac (**i**) and H3K4me1 (**j**). **k**, **l** Motif analysis of differential enrichment loci of H3K27ac (**k**) and H3K4me1 (**l**). ∗∗∗∗*p* < 0.0001

To understand whether and how diet and obesity impact enhancers, promoters, and gene expression in colonic epithelium, we generated genome-wide maps for H3K4me1 and H3K27ac by chromatin immunoprecipitation sequencing (ChIP-seq) in colon epithelial cells. In sum, we identified ~ 45,000 and ~ 85,000 regions containing H3K27ac and H3K4me1 in both males and females. Regions with both H3K27ac and H3K4me1 markers are typically classified as active enhancers, while those carrying only H3K4me1 are poised enhancers. We merged the biological replicates in each group and found that there were nearly identical numbers of poised enhancers and active enhancers in both groups (Fig. 2a; Additional file 2: Figure S4a). A comparison of the overlap between active enhancers and active promoters in obese and lean mice indicated that differential enrichment was found more frequently in enhancer regions (82.2% overlap in males, 79.2% overlap in females) than in promoters (92.2% overlap in males, 90.8% overlap in females) (Fig. 2b; Additional file 2: Figure S4b).

To investigate the relationship between enhancer status and gene expression, we assigned each identified enhancer to the closest transcription start site (TSS), allowing a maximal distance of 10 kb. Genes associated with active enhancers show, on average, higher expression levels than genes associated with poised enhancers and higher than genes without any enhancers, in both male and females, in both HFD and LFD groups (Fig. 2c; Additional file 2: Figure S4c, e, g). Interestingly, expression levels generally increased with the number of active enhancers associated with a given gene (Fig. 2d; Additional file 2: Figure S4d, f, h), suggesting that enhancers act together to define the expression level of their target genes.

Since enhancer marks showed a strong correlation with gene expression, we next asked how obesity changed the host gene expression profile and epigenome. Using the Mouse Transcriptome gene chip, we found 134 and 68 differentially expressed transcripts in obese males and females, respectively, with a *p* value < 0. 001 [17] (Additional file 4: Table S3). We validated differentially expressed genes by qPCR (Additional file 2: Figure S5a–d). We previously found that obesity drives epigenomic alterations in colonic epithelium resembling cancer progression in mice when analyzing animals from a colony known to be positive for pathogens [18]. To understand whether the differentially expressed genes in obese mice with commensals may resemble changes evident in human colorectal cancer, we compared differentially expressed genes in our study to colorectal cancer data sets in Oncomine (http://oncomine.org). We evaluated the top ten genes that were most highly up- and downregulated in our study across both normal and colon cancer data sets; strikingly, the majority changed in the same direction in obese mice when comparing normal colon with colon mucinous adenocarcinoma (Fig. 2e; Additional file 2: Figure S4i) in both sexes.

Analysis with Diffbind [19] revealed nearly 2000 loci with significantly (FDR < 0.01, fold change > 2) increased or decreased ChIP signal for H3K27ac and H3K4me1 (Fig. 2f, g; Additional file 2: Figure S4j). As expected, these differentially enriched loci were mostly located far from TSS, and were classified as enhancers (Fig. 2h; Additional file 2: Figure S4k). Next, we asked whether those H3K27ac differentially enriched loci overlapped with H3K4me1; we found more than 85% were enriched with H3K4me1 in both males and females (Additional file 2: Figure S4l), suggesting that obesity-induced changes mainly occurred at enhancer loci that were already poised. Consistent with our previous study, Ingenuity Pathways Analysis (IPA) revealed a number of enriched pathways within genes near these loci, most of which were associated with gastrointestinal diseases, such as intestinal cancer (Fig. 2i, j; Additional file 2: Figure S4m, n).

Enhancer regions harbor transcription factors, which bind cognate *cis*-acting DNA sequences and enable selective gene expression and regulation. To explore differential transcription factor occupancy in our system, we used HOMER [20] to determine which transcription factor binding motifs were present in these differentially enriched loci. The most significantly enriched motifs (Fig. 2k, l; Additional file 2: Figure S4o, p) exhibited striking concordance across different histone marks, suggesting mechanistic similarities in the biological response to obesity. In H3K27ac enriched loci, the top five enriched motifs corresponded to the known consensus binding sequences for nuclear receptors (NR; HNF4α), basic leucine-zipper (bZIP; FRA1, JUN-AP1 and FOSL2), and zinc finger (ZF; EKLF) family transcription factors in males, and to sequences for binding of ZF (EKLF) and bZIP (FOSL2, JUN-AP1, FRA1, and ATF3) in females (Fig. 2k; Additional file 2: Figure S4o). At H3K4me1 enriched loci, we also found that motifs for the NR, bZIP, and ZF families were highly enriched in both males and females (Fig. 2l; Additional file 2: Figure S4p).

### Diet and obesity affected HNF4α binding in colon epithelium

**Fig. 3:**
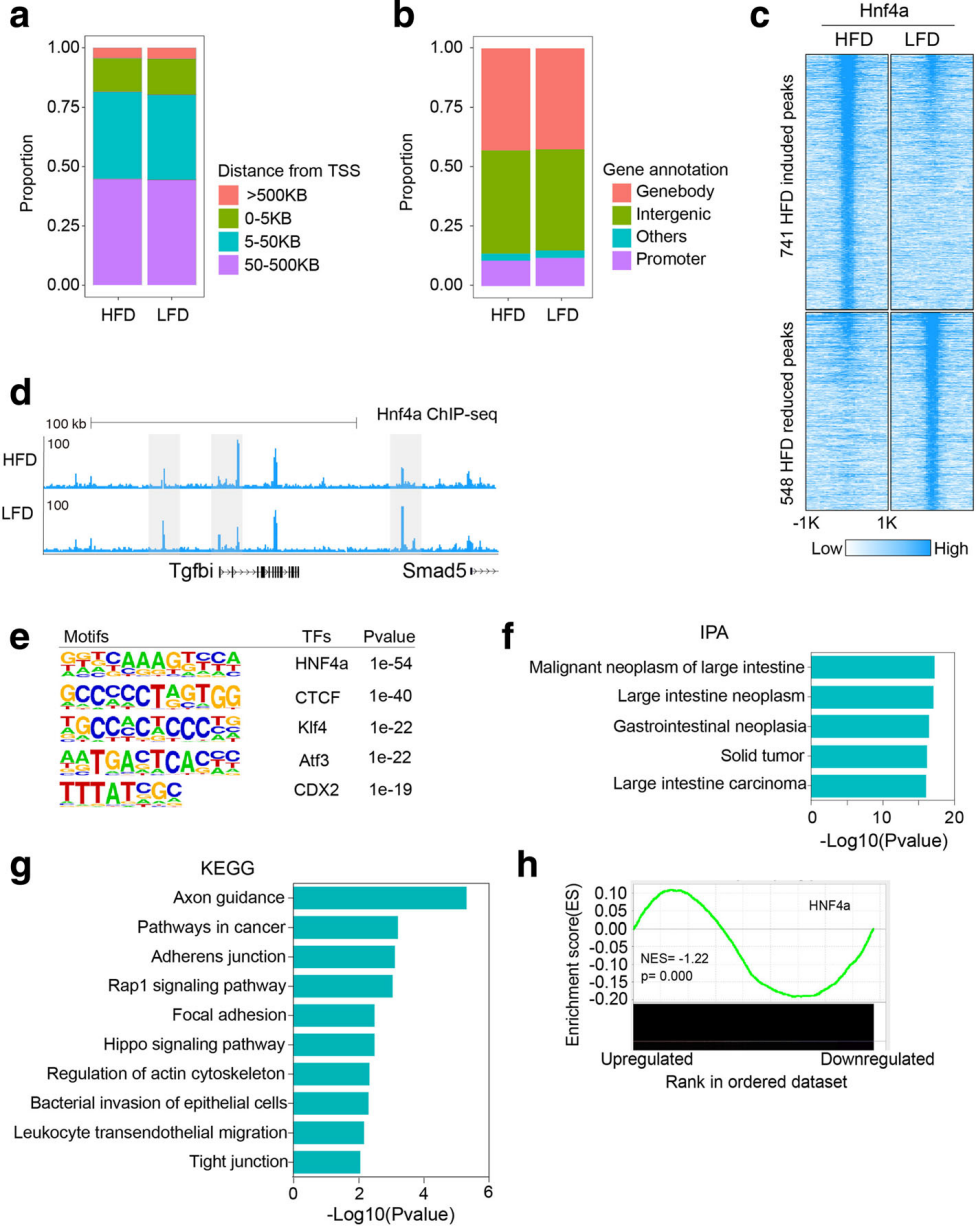
Diet and obesity altered HNF4α binding in colon epithelium. **a** Proportion of genes with different distances from HNF4α binding sites to TSS. **b** HNF4α binding site distribution in the genome. **c** Heatmap of differential HNF4α enrichment in colonic epithelium as a function of diet. **d** Representative genome browser shot of differentially enriched loci. **e** Motif analysis of differential enrichment of HNF4α. **f** IPA analysis of differential enrichment of Hnf4α. **g** KEGG analysis of different enrichment of HNF4α in HFD and LFD groups. **h** GSEA analysis of target gene sets of HNF4α

To understand whether diet and obesity can affect the distribution of a model signal responsive transcription factor, we carried out ChIP-seq for the nuclear receptor HNF4α in lean and obese male mice (Fig. 3a–h). In total, we identified 21,594 HNF4α binding sites in the control diet (LFD) group. To understand the genome-wide distribution of HNF4α, we compared binding sites with transcriptional regulatory sequences and found that most sites were far from TSS and localized in intergenic regions (Fig. 3a, b). Compared to the control diet (LFD) group, HNF4α has similar genome-wide localization in obese animals, although there was a modest decrease in the number of binding events (18,658 binding sites; ~ 15% decline). Although the majority of HNF4α-enriched loci are unaffected by diet, we did identify 1289 binding sites with differential enrichment (Fig. 3c, d). Motif analysis showed that factors including CDX2, known to coregulate genes in colon involved in lipid uptake and metabolism [21], were enriched at differentially occupied HNF4α-bound loci (Fig. 3e).

IPA analysis showed that genes near these differential binding sites were enriched in a number of pathways associated with gastrointestinal diseases, including colon cancer (Fig. 3f). KEGG analysis also showed that genes with differential HNF4α binding were enriched in pathways that are integral to maintenance of the homeostasis of the intestine (Fig. 3g). Integration of HNF4α ChIP-seq and gene expression data demonstrated that genes with HNF4α binding sites were enriched in the genes downregulated in the HFD group compared to the LFD group (Fig. 3h). These data suggest that a subset of the transcriptional program downstream of HNF4α is reprogrammed by obesity and the accompanying alterations in microbiome.

### Gut microbiota transplantation induced a pre-obesity phenotype

**Fig. 4:**
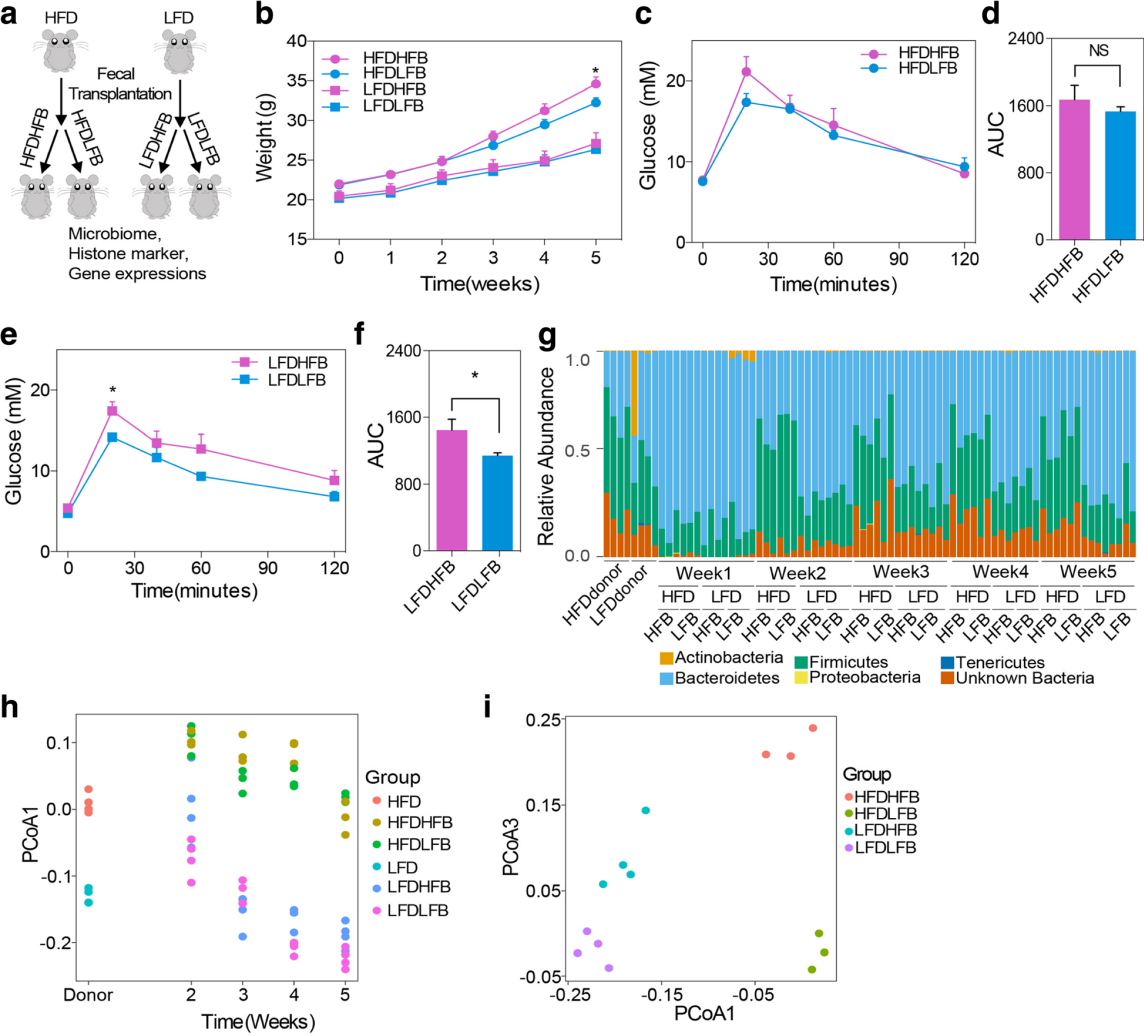
Bacteria transplantation induced a pre-obese phenotype in mice. **a** Study design of bacterial transplantation. **b** Weights of germ-free mice on either HFD or LFD that have been transplanted with either high fat bacteria (*HFB*) or low fat bacteria (*LFB*). **c**, **d** Time course and area under the curve (*AUC*) for IPGTT of mice in HFDHFB and HFDLFB groups. **e**, **f** Time course and AUC for IPGTT of mice in LFDHFB and LFDLFB groups. **g** Relative abundance of bacteria at phylum level in mice for 5 weeks with different bacterial transplantation and diets. **h** PCoA of unweighted Unifrac distances over time for bacteria from animals with different bacteria and diets. **i** PCoA of unweighted Unifrac distances at week 5 for mice with different bacteria and diets. Data are presented as mean ± standard error of the mean. n = 7–8 per group (weight and IPGTT) or 3–4 per group (16s sequencing). ∗*p* < 0.05; *NS* non-significant

To decipher the relationships between gut microbiome, diet, and host epigenome, we carried out microbiota transplantation/diet studies. Fecal samples prepared fresh from male donor mice fed the LFD or HFD were introduced into 6-week-old male and female germ-free mice fed either the LFD or the HFD (Fig. 4a). Interestingly, we found that male recipient mice fed the obesogenic (HFD) diet and receiving bacterial transfer from obese animals (HFDHFB) gained more weight than those receiving bacteria from controls (HFDLFB) (Fig. 4b), while female recipient mice did not (Additional file 2: Figure S6a). This difference was not seen in groups on the control diet, suggesting that the combination of bacterial transfer from obese animals and the obesogenic diet has a compound and sex-specific effect on the host. We also performed a glucose tolerance test and found blood glucose levels were slightly higher in animals on the control diet that received bacterial transfer from obese animals (the LFDHFB group; Fig. 4c–f). These observations indicate that the microbiome–diet interaction, presumably through bacterial metabolites produced from the host diet, induces metabolic changes and/or weight gain in the host.

Next, we checked the microbiota composition in each group by 16S rDNA sequencing (Fig. 4g; Additional file 5: Table S4). It took nearly one week for bacteria to colonize the gut of recipient mice and analysis of the microbiomes from donor and recipient fecal samples revealed that the recipients all exhibited a consistent shift in microbial diversity that was affected by the diets (Fig. 4g, h; Additional file 2: Figure S6b). In female animals, the bacterial response to diet, as evidenced by the ratio of *Bacteroidetes* to *Firmicutes*, was substantially different than in males (Additional file 2: Figure S6b). Although the recipients’ gut microbiome was shaped dramatically by diet, there remained a difference between those receiving bacteria from obese donors versus those receiving bacteria from control donors (Fig. 4i, j; Additional file 2: Figure S6c).

### Gut microbiota remodel the host epigenome and transcriptome

**Fig. 5:**
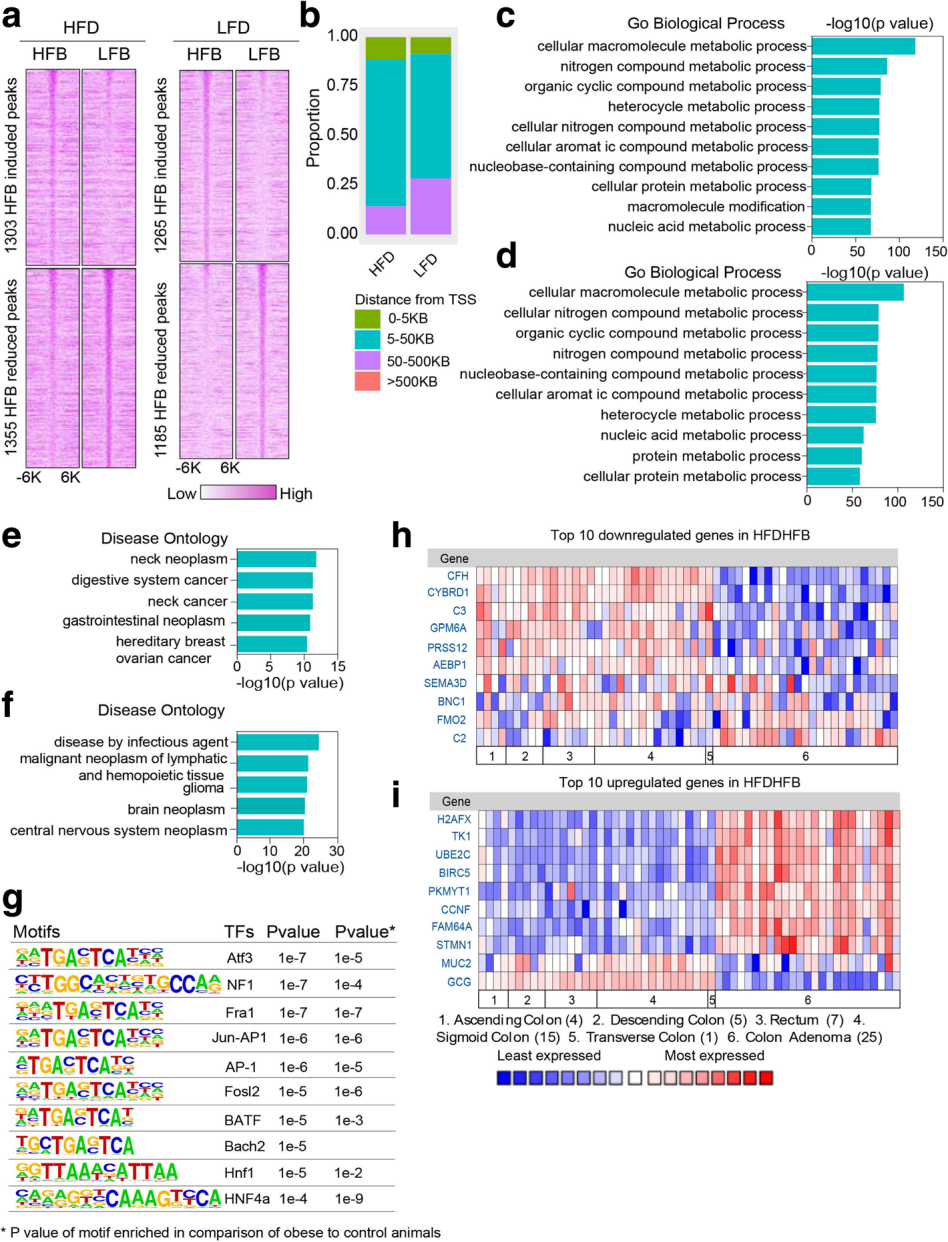
Gene ontology analysis of differentially enriched and expressed genes following manipulation of microbiota and diet. **a** Heatmap of differentially enriched loci for H3K27ac in HFDHFB, HFDLFB, LFDHFB, and LFDLFB groups. **b** Distance of differentially enriched loci from the nearest TSS. **c**, **d** GO analysis of loci with differential enrichment of H3K27ac in HFDHFB and HFDLFB groups (**c**) and LFDHFB and LFDLFB groups (**d**). **e**, **f** Disease ontology analysis in HFDHFB and HFDLFB groups (**e**) and LFDHFB and LFDLFB groups (**f**). **g** Motif analysis of loci with differential enrichment of H3K27ac in HFDHFB and HFDLFB groups (combined). **h**, **i** Oncomine analysis of differentially expressed genes in HFDHFB and HFDLFB groups

To characterize the relationship between bacterial status, diet, and active enhancers, we profiled H3K27ac in colon epithelium using ChIP-seq in the animals receiving microbiome transfer. In total, we found ~ 45,000 peaks in both diet conditions. In mice fed the obesogenic diet (HFD), we found the transplantation of bacteria from obese donors (HFB) induced an increase in H3K27ac marks at 1303 loci and a reduction at 1355 loci when compared to animals on the same diet that received bacteria from control donors. In mice fed the control diet (LFD), bacterial transfer from obese donor animals (HFB) induced an increase at 1265 loci and a reduction at 1185 loci compared to animals on the same diet that received bacterial transfer from control donors (LFB) (Fig. 5a). As expected, differentially enriched loci were far from TSS and were designated as enhancers (Fig. 5b). Using GREAT [22] to annotate the functions of these loci, we found that these loci with altered chromatin features resulting from differences in bacterial donor exhibited an enrichment in metabolism-related pathways regardless of host diet (Fig. 5c, d). This demonstrates that in animals on the same diet, altering microbiota affects host metabolic pathways. Disease ontology analysis showed that genes associated with differentially enriched loci in mice fed the obesogenic diet (HFD) have similar profiles to gene sets expressed in digestive system cancers and gastrointestinal neoplasms. Differentially enriched loci from mice fed the control diet (LFD) were associated most strongly with disease by infectious agent and with genes implicated in diverse conditions not specific to gastrointestinal disease (Fig. 5e, f).

Transcription factor motifs underlying these bacteria-dependent differentially acetylated loci were investigated using HOMER [20]. In animals on the obesogenic diet, the differentially acetylated loci were enriched in binding sites for the leucine zipper factor ATF3b and the nuclear receptor HNF4α (Fig. 5g). We chose several H3K27ac differentially enriched loci and assessed HNF4α binding by ChIP-pcr. These results were consist with the ChIP-seq analysis conducted on animals on a long-term dietary regimen (Additional file 2: Figure S7a). A large majority of the K27Ac-enriched motifs overlapped with those found enriched when comparing obese and control animals (Fig. 2k, l).

We next wanted to ascertain how gene expression profiles correlated with epigenetic changes. RNA-seq analysis revealed that animals on the obesogenic diet with different bacterial donor sources differed (*p* < 0.001; Additional file 6: Table S5) [23] at 166 genes (HFDHFB vs HFDLFB). In contrast, on a control diet, only 17 genes had expression changes dependent on the microbiome (LFDHFB vs LFDLFB; Additional file 6: Table S5) [23]. We validated differentially expressed genes in HFDHFB/HFDLFB by qPCR (Additional file 2: Figure S5e, f). Since the HFDHFB remodeled the epigenome with some similarities to pathways related to colon cancer, we asked whether changes in the gene expression profile did so as well. To examine potential similarities, we compared differentially expressed genes in our study to colorectal cancer data sets in Oncomine (http://oncomine.org). We evaluated the top ten genes most highly up- and downregulated in our study across normal and colon cancer data sets (Fig. 5h, i); the majority were changed in the same direction in the HFDHFB group when comparing normal colon with colon adenoma (Fig. 5h, i). These findings indicated that the combination of HFD and high fat bacteria can induce a gene expression profile that has partial resemblance to that observed in human colorectal cancer.

**Fig. 6:**
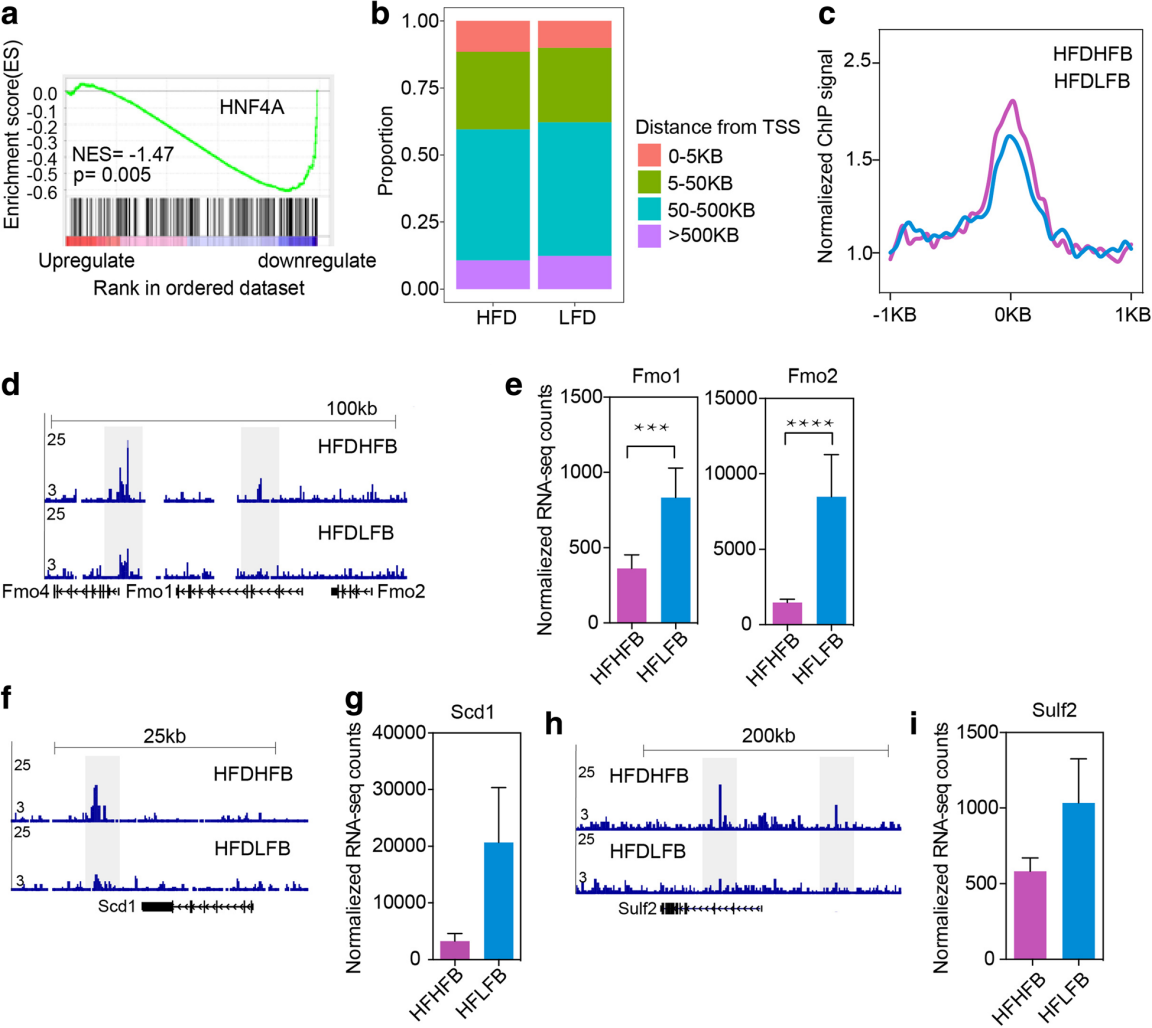
Regulatory role of HNF4α in colon epithelium. **a** GSEA analysis of target gene sets of HNF4α. **b** Distance of HNF4α binding sites to the nearest TSS. **c** The average, normalized ChIP-seq signal in HFDHFB and HFDLFB groups at gained HNF4α binding sites that were identified in HFD and LFD groups. **d** Genome browser tracks showing HNF4α occupancy near *Fmo1* and *Fmo2*. **e** Normalized counts of transcripts for *Fmo1* and *Fmo2* in HFDHFB and HFDLFB groups. **f** Genome browser tracks showing HNF4α occupancy near the *Scd1* locus. **g** Normalized counts of transcripts for *Scd1* in HFDHFB and HFDLFB groups. **h** Genome browser tracks showing HNF4α occupancy near *Sulf2*. **i** Normalized counts of transcripts for *Sulf2* in HFDHFB and HFDLFB groups. *P* value from DESeq2, ∗*p* < 0.05; ∗∗*p* < 0.01; ∗∗∗*p* < 0.001; ∗∗∗∗*p* < 0.0001

To investigate whether the gene expression profile is reflective of upstream transcription factor changes, we performed GSEA and found that genes regulated by the nuclear receptor HNF4α were enriched in the genes downregulated in the HFDHFB group compared to the HFDLFB group (Fig. 6a). HNF4α, which is a signal responsive transcription factor, has an important role in maintaining intestinal homeostasis in response to microbiota [24]. Transcription factor motif analysis of the subset of loci with less acetylation in animals with bacteria from an obese donor also indicated enrichment for HNF4α (Additional file 2: Figure S7b, c). HNF4α was reported to recruit co-repressors to inhibit gene expression integral to lipid homeostasis in liver [25]. To understand the regulatory role of HNF4α in our study, we carried out ChIP-seq in the HFDHFB and HFDLDB groups. As expected, HNF4α binding sites were located far from TSS (Fig. 6b). Comparison of HNF4α ChIP-seq data collected from chronic HFD and LFD mice demonstrated that loci gaining HNF4α binding in chronic HFD also had increased signal in the HFDHFB group (Fig. 6c).

Through integrated analysis of HNF4α ChIP-seq data and RNA-seq data at exemplar genes, we determined that genes gaining HNF4α binding were downregulated in HFDHFB (Fig. 6d–i). We observed increased binding at the *Fmo1/2* locus (Fig. 6d–e) that correlated with decreased expression of these genes in animals on the obesogenic diet receiving bacterial transfer from obese animals (HFDHFB). FMO1 and FMO2 play important roles in iron metabolism and regulate formation of reactive oxygen species [26]. Genetic deletion of *Hnf4α* in mice decreased binding at the *Fmo 1/2* locus and increased their expression levels [26]. Likewise, we observed accumulation of HNF4α at the *Scd1* locus concomitant with decreased expression (Fig. 6f, g). SCD1, a key enzyme for fatty acid synthesis, was downregulated in the liver under fasting conditions which increased HNF4α level, correlating with decreased SCD1 expression [27]. Finally, we observed increased accumulation of HNF4α at the *Sulf2* locus along with decreased expression (Fig. 6 h, i). *Sulf2* expression has previously been reported by others to have a strong inverse correlation with HNF4α [28]. Therefore, these data strongly suggest that bacterial metabolism of the host diet has the capacity to influence host transcription factor action and regulation of gene expression.

## Discussion

Obesity and obesity-related conditions are major health problems worldwide, underscoring the importance of elucidating the etiology of metabolic diseases and development of targeted therapies or preventative measures. However, given the complex interplay of host genetics with environment (diet) and the symbiotic microbiota, it is challenging to identify and enumerate primary causes. There is growing evidence that dysbiosis of the gut microbiota is linked to the pathophysiology of obesity [11]. Clarity on molecular mechanisms by which bacteria in the intestinal lumen can impact the host in such profound ways is beginning to emerge. Gut microbiota was reported to participate in regulating gene expression through DNA methylation in intestinal epithelial cells. Those DNA methylation changes were Dnmt1 independent and can be recapitulated by fecal transplantation [29]. Besides epithelial cells, gut microbiota modified open chromatin status in intestinal intraepithelial lymphocytes [30]. Gut microbiota can regulate chromatin features in multiple host tissues in a diet-dependent manner resulting in alterations in host physiology [3] and gene expression, highlighting the potential for epigenetic effects to underlie a subset of the host outcomes linked to the microbiome.

In this study, we explored how the host epigenome responds to diet and microbiome using a murine diet-induced obesity model with normal and germ-free mice. We observed that lean and obese animals have very different microbial profiles as previously reported by others [11]. Kinetic analysis of the evolution of the microbiome over time on an obesogenic diet revealed that the diet has a dominant role in shaping the bacterial profile. Prior to the development of the murine equivalent of obesity [16], we observed microbiome evolution towards a profile resembling that found in extreme obesity (Fig. 1e–h). However, female mice respond to the diet more slowly than the male mice and had a different pattern of weight gain (Additional file 2: Figure S1a), consistent with hormonal impacts on the microbiome [31]. Long-term adaptation to an obesogenic diet in females results in microbiome composition more similar to males. The modified microbiome, in turn, promotes weight gain and metabolic dysregulation in the host (Fig. 4b–f) and creates a feed-forward loop that promotes obesity.

What is the consequence of these obesity-associated microbiota changes? Remodeling of the microbiome towards that of obesity impacts disease risk. The obesogenic diet resulted in decreased abundance of specific bacterial groups, including *Bifidobacteria* (Fig. 1a), which have beneficial actions on the host. *Bifidobacteria* can regulate tight junction protein expression, reduce proinflammatory cytokines in mucus, and maintain the epithelial barrier [32]. A reduction in “beneficial” flora is associated with onset of chronic inflammation, a hallmark predisposing factor for cancer [33]. Along these lines, obese animals and germ-free animals reconstituted with microbiota from obese donors elaborated epigenetic alterations at loci with connections to cancer (Figs. 2i, j and 5e). Our current findings reinforce our previous study suggesting links between obesity and increased colorectal cancer risk through epigenetic alterations [18]. They also add an additional complexity—the epigenetic changes documented in both studies likely result not from obesity, but from the metabolism of an obesogenic diet by a specific microbiota. We find that neither the obesogenic diet nor the microbiome of an obese animal is sufficient to program the epigenome of colonic epithelia. Both factors are required, strongly suggesting that metabolites produced by bacteria from the host diet constitute signals to the host epigenome, potentially serving to alter gene expression programs more efficiently than either factor alone.

Action on the host epigenome is possible through conventional signaling pathways or through direct effects on transcription factors. In our studies, we observed epigenetic alterations at binding sites for signal responsive transcription factors and nuclear receptors (Figs. 2k, l and 5g), prime targets for signaling from microbial products. We found obesity can affect HNF4α bindng and reprogram gene expression in colon epithelium (Fig. 3f–h). HNF4α is an orphan nuclear receptor involved in metabolic regulation with the potential to both activate and repress transcription [25, 34]. In colitis, HNF4α was considered protective against inflammation and genes downregulated in colitis were enriched in HNF4α binding sites [35]. In our study, genes downregulated in obesity were enriched in HNF4α binding sites and HNF4α levels were slightly higher in obese animals at these sites. HNF4α was recently found to recruit a co-repressor to downregulate gene expression [25]. In our studies, genes gaining HNF4α binding were downregulated in the HFDHFB group, including genes with potential relevance to disease such as *Fmo1*, *Fmo2*, *Scd1*, and *Sulf2* [36–39]. Collectively, these data suggest a regulatory role for HNF4α in gene expression that is influenced by diet and bacteria. Along these lines, the identification of lipids as ligands for HNF4α [40] are consistent with the possibility that bacterial metabolism of diet produces fatty acids that serve as ligands for HNF4α.

## Conclusions

Our results highlight potential interactions between host diet and microbiome and their effects on the host epigenome, which prime enhancers in the host colon epithelium for obesity and obesity-related conditions. These findings provide new insights into host–microbiota interactions with potential relevance to obesity and obesity-related diseases.

## Methods

### Mice

Five-week-old C57BL/6 male and female mice were purchased from Jackson Laboratory and acclimated at NIEHS for one additional week. Mice were singly housed and placed on either a 10% fat diet (LFD) or a 60% fat diet (HFD) (D12450B and D12492, respectively; Research Diets) for up to 20 weeks in a specific pathogen-free animal facility. Five-week-old C57BL/6 male and female germ-free mice were purchased from Taconic and used for microbiota transplantation. For these fecal transplantation experiments, ~ 100 mg stool was collected fresh in the morning from male donor mice already on the HFD/LFD and re-suspended in 300 μl phosphate buffered saline (PBS), homogenized, and centrifuged (300 x g, 3 min) to remove debris. Recipient mice were administered 100 μl of the supernatant by oral gavage four times a week for 5 weeks. All animal experiments were approved by the NIEHS Institutional Animal Care and Use Committee and were performed according to the NRC Guide for the Care and Use of Laboratory Animals.

### 16S rRNA gene sequencing and data analysis

Stool samples were collected fresh from individual mice and stored at −80 °C until DNA isolation. Fecal bacterial DNA was extracted using a QIAamp DNA Stool mini kit (Qiagen) according to the manufacturer’s instructions. Two rounds of PCR reactions were used to amplify the V3 region of the bacterial 16S rRNA gene for sequencing. Primers used in the first round of PCR contained the overhang sequences with Illumina adapters; forward primer, TCGTCGGCAGCGTCAGATGTGTATAAGAGACAGCCAGACTCCTACGGGAGGCAG; reverse primer, GTCTCGTGGGCTCGGAGATGTGTATAAGAGACAGCGTATTACCGCGGCTGCTG. PCR conditions were 98 °C for 3 min; 15 cycles of 98 °C for 30 s, 62 °C for 30 s, and 72 °C for 30 s. A second round of PCR was used to add the index to the amplicons for sequencing. PCR conditions were 95 °C for 3 min; 8 cycles of 95 °C for 30 s, 55 °C for 30 s, and 72 °C for 30 s. Sequencing was performed on the MiSeq platform in multiplex. Amplicons spanning the variable region 3 of the 16S rRNA gene were sequenced and analyzed by Mothur [41]. After quality control and trimming the adaptors, paired-end reads were joined and mapped to the Greengenes 13.8 release database. Operational taxonomic units (OTUs) were picked against the Greengenes database, using a 97% similarity threshold. To adjust for differences in sequencing depth, all samples were normalized to the same number in the following analysis. Lefse was used to compare the differential bacterial abundance in the HFD and LFD groups with default settings [14].

### Measurement of short chain fatty acids

Fecal samples (50–150 mg each) were homogenized in 1 mL 0.005 M NaOH containing an internal-standard solution and centrifuged at 4 °C (3000 x g, 10 min). Supernatant (0.5 mL) was transferred to glass tubes followed by the addition of 0.3 mL water, 0.5 mL n-propanol:pyridine mixture (3:2, v/v), and 0.1 mL propyl-chloroformate. After derivatization, samples were extracted by a two-step procedure with hexane. Sodium sulfate was added to remove traces of water from hexane prior to GC-MS analysis [42].

### Colon epithelial cell isolation

Colonic epithelial cells were isolated and fixed as described previously [18]. Briefly, mice were terminally euthanized and the colons were harvest in a petri dish on ice. Then the colon tissue was opened longitudinally and flushed with cold PBS. The colons were cut into ~ 5-mm fragments and placed into 50 ml conical tubes that were filled with 30 ml of cold PBS (Mg/Ca free)/EDTA (5 mM). The fragmented colon tissues were shaken for 20–30 min until most epithelial cells were sloughed. Lamina propria was removed and the epithelium cells were pelleted by 500g for 5 min. After two washes with cold PBS, cells were collected and reserved for following studies. For purity test, isolated epithelium cells were digested to single cell solution by TrypLE Express (Invitrogen) and stained with EpCAM-AF647 (Biolegend) and CD45-PE (Biolegend) according to the instructions. The purity of the isolated epithelium cells was around 85% (Additional file 2: Figure S8).

### ChIP-Seq,ChIP-pcr, and data analysis

Epithelial cells were treated with 1% formaldehyde in PBS for 10 min at room temperature. Cross-linking was terminated by addition of glycine. Cells were lysed in buffer A (1% SDS, 5 mM EDTA, 50 mM Tris-HCl (pH 8.1), protease inhibitor cocktail) and sonicated using a Bioruptor (Diagenode) to generate ~ 300-bp fragments for immunoprecipitation. The collected supernatant was diluted 10× with dilution buffer (1% Triton X-100, 2 mM EDTA, 150 mM NaCl, 20 mM Tris-HCl (pH 8.1), protease inhibitor cocktail). The chromatin (4 μg) was subjected to immunoprecipitation with 1 μg of H3K27ac (ab4729, Abcam), H3K4me1 (ab8895, Abcam), HNF4α (ab41898) antibody and incubated overnight. The samples were incubated with either protein A or G (EMD Millipore) beads for 1 h. The beads were washed with the following buffers: low salt (0.1% SDS, 1% Triton X-100, 2 mM EDTA, 20 mM Tris-HCl, pH 8.1, 150 mM NaCl), high salt (0.1% SDS, 1% Triton X-100, 2 mM EDTA, 20 mM Tris-HCl, pH 8.1, 500 mM NaCl), LiCl (0.25 M LiCl, 1% NP-40, 1% deoxycholate, 1 mM EDTA, 10 mM Tris.HCl, pH 8.1) and then twice with TE buffer. The protein–DNA complexes were eluted from the beads with 100 μl elution buffer (1 mM DTT, 1% SDS, 100 mM NaHCO3) and reverse crosslinked at 65 °C for 4 h with Proteinase K. ChIPed DNA was purified by AMPure XP beads (Beckman). For ChIP-qPCR, primers were listed in Additional file 7: Table S6. For ChIP-seq, 2 ng of ChIPed DNA was prepared using NEXTflex Rapid Illumina DNA-Seq Library Prep Kit (Bio Scientific). The resulting libraries were sequenced on Illumina Nextseq 500 as 35-bp paired ends.

Raw reads were filtered by quality score and aligned to the mouse genome (mm9). Unique aligned and de-duplicated reads were used for peak calling using SICER (v1.1) [43]. The parameters for H3K27ac and H3K4me1 were size 200, gap size 200, fragment size 200, FDR cutoff 0.001, and size 200, gap size 400, fragment size 200, and FDR cutoff 0.001, respectively. For overlap analysis of active enhancers and active promoters, biological replicates were merged and normalized to 30 million reads per group. Differentially enriched H3K27ac and H3K4me1 loci were identified using the diffBind package and results were filtered with *p* values < 0.01 and fold change > 2. The HOMER package [20] was used to do the peak calling for Hnf4α with default settings. Motifs were extracted from the differential H3K27ac, H3K4me1, and Hnf4α regions against a large set of randomly selected genomic fragments of the same size by HOMER. Gene annotations were also generated by HOMER packages.

### Gene arrays, RNA-seq, and data analysis

The Mouse Transcriptome Assay 1.0 was used to profile gene expression in colon epithelium of mice fed either the HFD or LFD for 20 weeks. The feature extractor processed signal was log2 transformed by the Partek Genome Suite. ANOVA was used to identify the differentially expressed genes with *p* values < 0.001.

For the transplantation study, cells were scraped from the colon tissue and RNA was extracted by RNeasy Mini Kit (Qiagen). RNA-sequencing was done in Expression Analysis and sequence data were processed with STAR [44] to generate read alignments with mm9. Raw read counts for annotated genes were obtained with ‘featureCounts’ [45], normalized and analyzed using DEseq2 [46]. *P* value < 0.001 was used to identify the differentially expressed genes. Parts of the differentially expressed genes were validated by qPCR and primers are listed in Table S6. We applied gene set enrichment analysis (GSEA) to RNA-seq data using transcription factor targets from the Molecular Signatures Database (MSigDB). All genes in RNA-seq data were used and ranked according to the fold change multiple *p* value.

### Statistical analysis

All statistical analyses were performed by two-tailed Student's t test using GraphPad Prism7 (San Diego, CA, USA). The level of significance was set at *p* < 0.05; ∗*p* < 0.05; ∗∗*p* < 0.01; ∗∗∗*p* < 0.001; ∗∗∗∗*p* < 0.0001. All data are expressed as means ± standard error of the mean.

## Additional files

Additional file 1: Table S1. This file contains Table S1 which provides detailed descriptions of the components of the diets used in this study. (XLSX 9 kb)

Additional file 2: Figures S1–S8. Along with figure legends. (PDF 2613 kb)

Additional file 3: Table S2. This file contains tables providing bacterial abundance for male and female mice across the time points and diets utilized in the study. (XLSX 94 kb)

Additional file 4: Table S3. This file contains a table providing the *p* value and fold change for the Affymetrix gene expression microarray experiment performed on animals at 20 weeks of dietary treatment. (XLSX 4268 kb)

Additional file 5: Table S4. This Excel table provides the relative abundance of microbiota in donor mice and in recipient mice after bacterial transfer across the time points, diets, and transfer strategies outlined in the text. (XLSX 67 kb)

Additional file 6: Table S5. This Excel spreadsheet provides fold change, *p* values, and adjusted *p* values for the RNAseq data described in the text. Fold change and statistical significance are given for the comparisons within dietary groups across bacterial transfer. (XLSX 2254 kb)

Additional file 7: Table S6. The table presents the sequences of primer sets used for ChIP-PCR. (XLSX 35 kb)
