# Supplementary material for: An obesity-associated gut microbiome reprograms the intestinal epigenome and leads to altered colonic gene expression
Source: Genome Biol. 2018 Jan 23;19:7. doi: 10.1186/s13059-018-1389-1 (PMC5782396; doi:10.1186/s13059-018-1389-1)
Supplement: Supplementary file 2 — Along with figure legends. (PDF 2613 kb) [file 13059_2018_1389_MOESM2_ESM.pdf]

FigS1

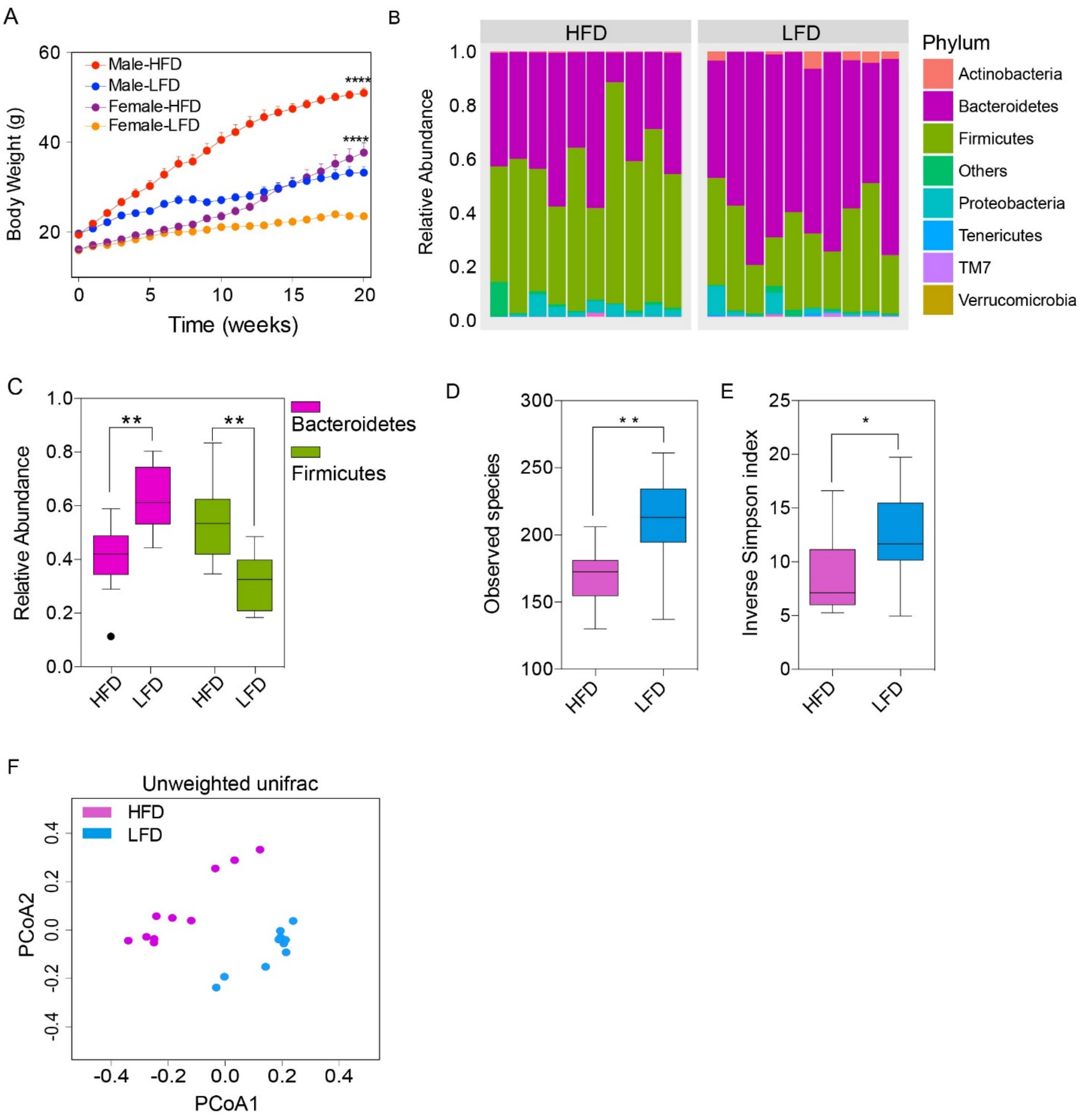

FigS1 High fat diet shaped host microbiota in male mice

(A) Weight of mice on HFD and LFD. (B) Relative abundance of bacteria at phylum level in male mice fed a HFD or LFD for 20 weeks. (C) Box plot of the two dominant bacterial phyla in HFD and LFD groups in Panel A. (D-E) Alpha diversity based on OTUs (observed species and inverse simpson index) in HFD and LFD groups in male mice. (F) Principal coordinate analysis

(PCoA) of unweighted Unifrac distances after 20 weeks on different diets. Data are presented as mean ± SEM. n = 9-10 per group. $*p < 0.05$; $**p < 0.01$; $***p < 0.001$; $****p < 0.0001$.

Fig S2

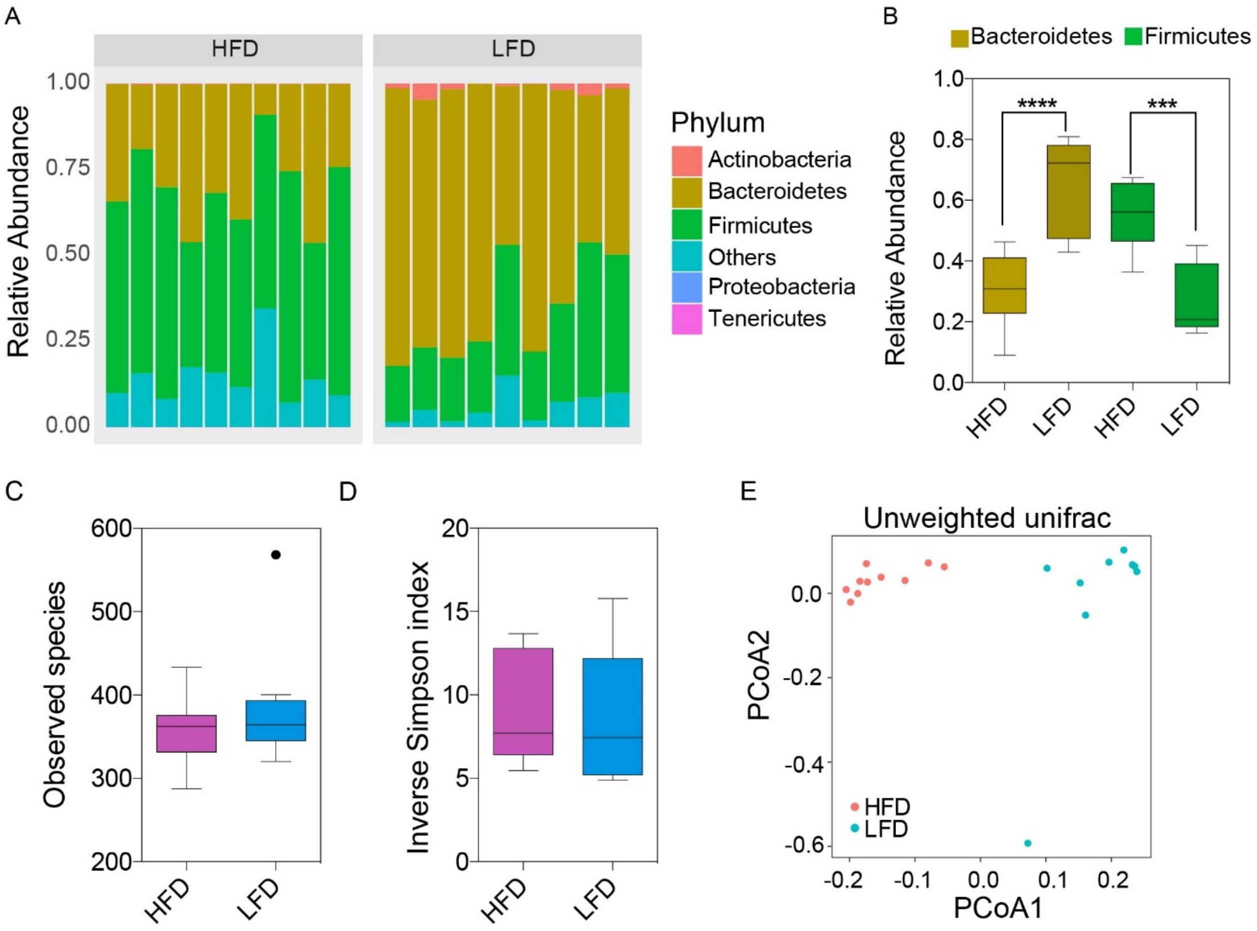

FigS2 High fat diet shaped host microbiota in female mice

(A) Relative abundance of bacteria at phylum level in female mice fed a HFD or LFD for 20 weeks. (B) Box plot of the two dominant bacterial phyla in HFD and LFD groups in Panel A. (C-D) Alpha diversity based on OTUs (observed species and inverse simpson index) in HFD and LFD groups in male mice. (E) Principal coordinate analysis (PCoA) of unweighted Unifrac distances after 20 weeks on different diets. Data are presented as mean ± SEM. n = 9-10 per group. ∗p < 0.05; ∗∗p < 0.01; ∗∗∗p < 0.001; ∗∗∗∗p < 0.0001.

Fig S3

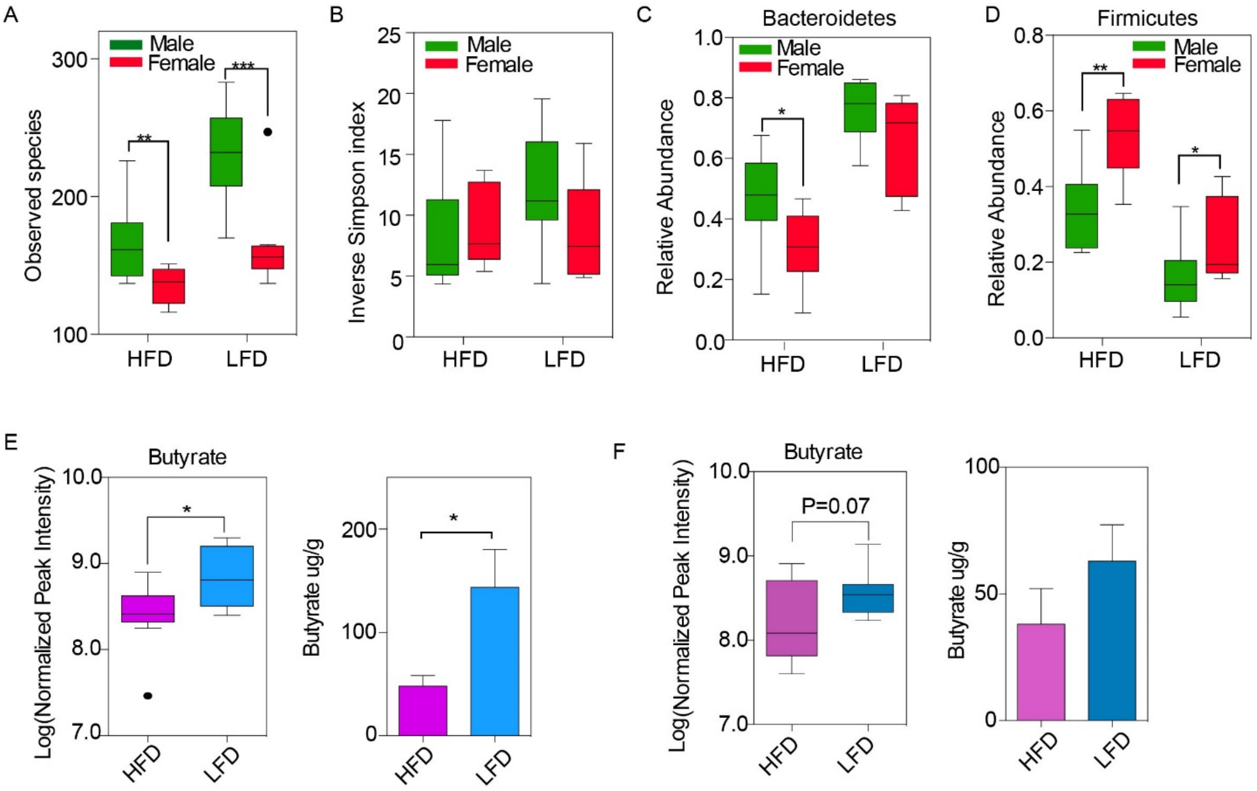

FigS3 Sex specific microbime and SCFAs in obese mice

(A-B) Alpha diversity in HFD and LFD groups in male and female mice. (C-D) Box plot of the two dominant bacterial phyla in HFD and LFD groups in male and female mice. (E-F) SCFA levels determined by GC-MS in male and female mouse fecal samples fed different diets. n = 10 per group for SCFA analysis. Data are presented as mean ± SEM. n = 9-10 per group. * $p < 0.05$; ** $p < 0.01$; *** $p < 0.001$.

Fig S4

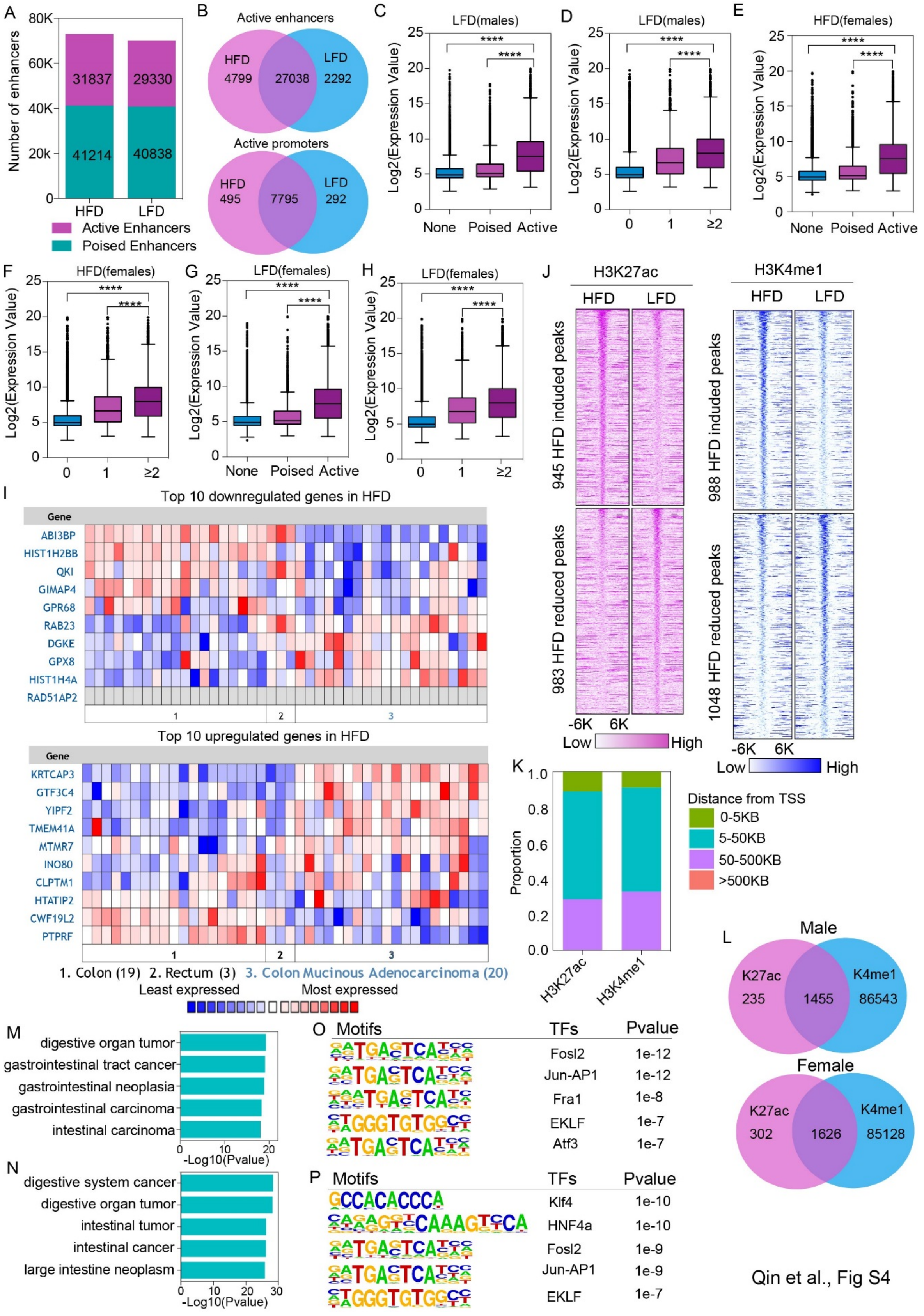

Qin et al., Fig S4

FigS4 Diet and obesity altered the host transcriptome and epigenome to resemble that found in colon adenocarcinoma in female mice

(A) Numbers of active (marked with both H3K27ac and H3K4me1) and poised enhancers (marked with H3K4me1) in obese and lean mice. (B) Overlap analysis of active enhancers and active promoters in obese and lean mice. (C) Expression levels at genes with none, poised or active enhancers in colonic epithelium from male mice on LFD. (D) Expression levels at genes with none, one, or more than one active enhancer in colonic epithelium in male mice on LFD. (E) Expression levels at genes with none, poised or active enhancers in colonic epithelium from female mice on HFD. (F) Expression levels at genes with none, one, or more than one active enhancer in colonic epithelium in female mice on HFD. (G) Expression levels at genes with none, poised or active enhancers in colonic epithelium from female mice on LFD. (H) Expression levels at genes with none, one, or more than one active enhancer in colonic epithelium in female mice on LFD. (I) Oncomine analysis of differentially expressed genes from animals on HFD compared to differentially regulated genes from normal colon, normal rectum and colon mucinous adenocarcinoma (groups 1, 2 and 3, respectively). (J) Heatmap of differential enrichment loci of H3K27ac and H3K4me1 from colonic epithelium in mice on different diets. (K) Proportion of genes with different distances from differential enrichment loci for H3K27ac and H3K4me1 to TSS. (L) Overlap analysis of differential enrichment loci of H3K27ac and H3K4me1. (M-N) IPA analysis of differential enrichment loci of H3K27ac (M) and H3K4me1 (N). (O-P) Motif analysis of differential enrichment loci of H3K27ac (O) and H3K4me1 (P). ****$p < 0.0001$.

Fig S5

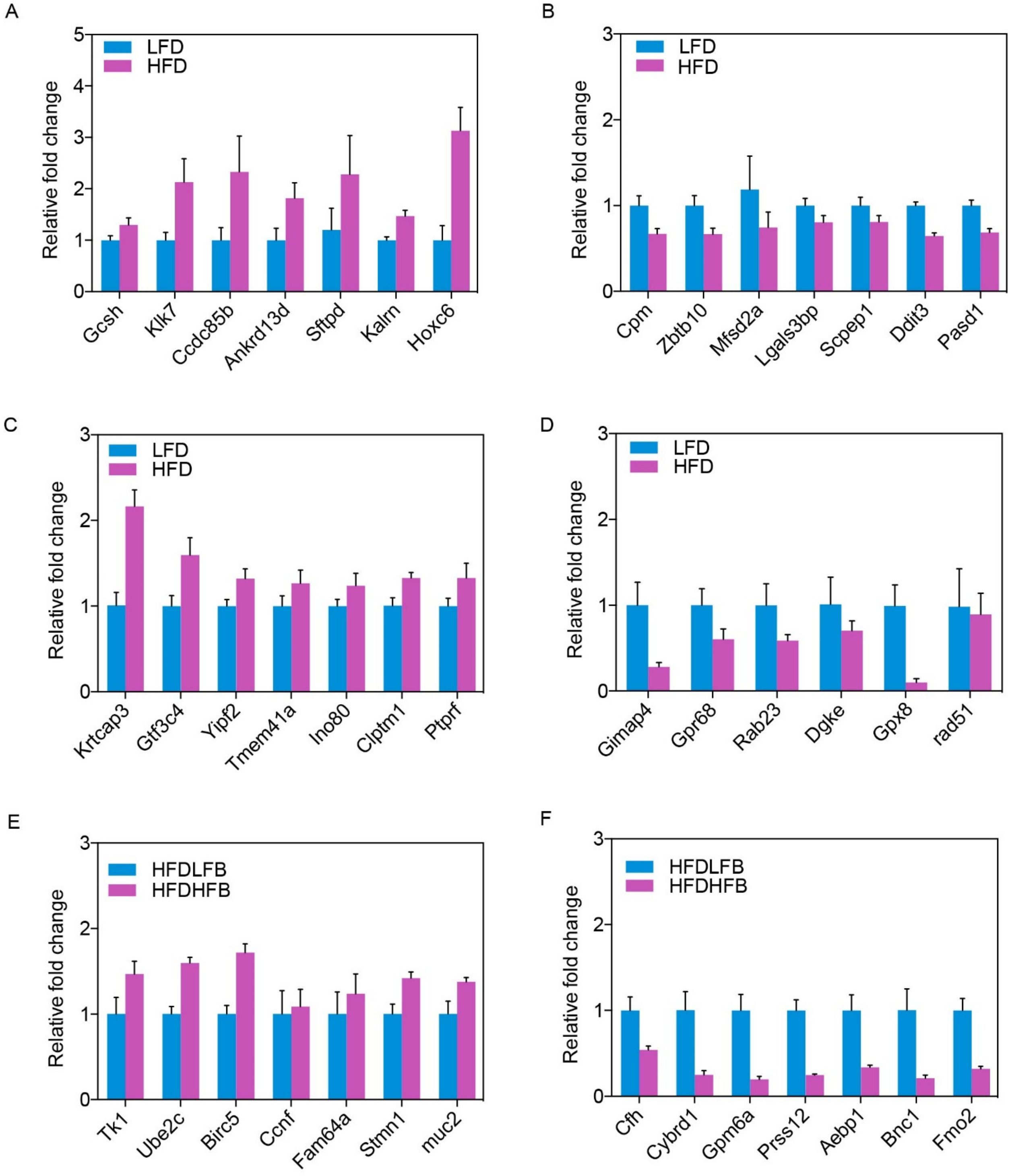

FigS5 Real time PCR validate the differential expressed genes

(A-B) Differential expressed genes indentified in males. (C-D) Differential expressed genes indentified in females. (E-F) Differential expressed genes indentified in male HFDHFB and

HFDLFB groups.

Fig S6

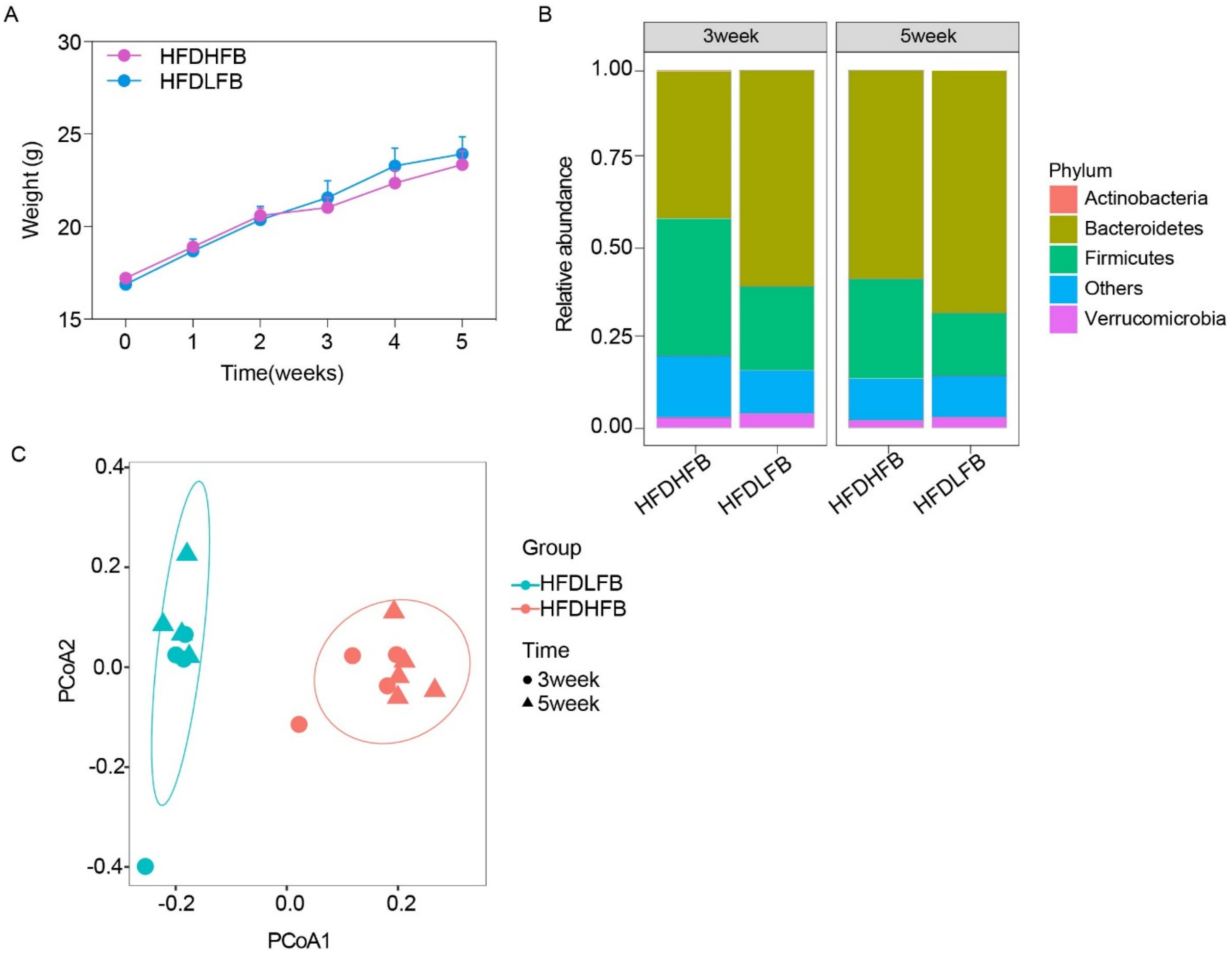

FigS6 Microbiome composition in female germ free mice.

(A) Weight of female germ free mice on HFDHFB and HFDLFB. (B) Relative abundance of bacteria at phylum level after transplantation for 3 and 5 weeks. (C) Principal coordinate analysis (PCoA) of Bray Curtis distances after 3 and 5 weeks transplantation.

Fig S7

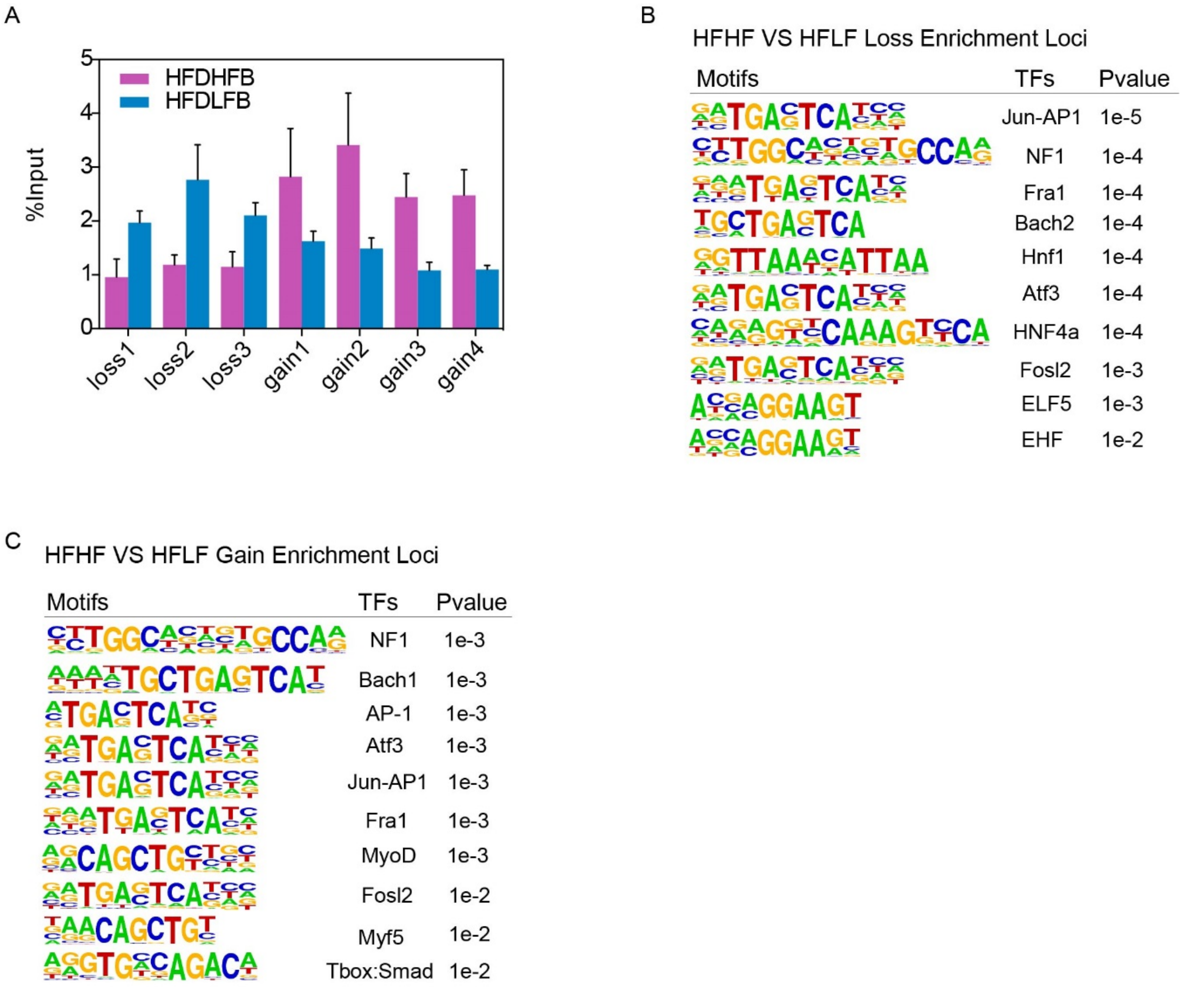

FigS7 Motif analysis of gain and loss enrichment loci of H3K27ac

(A)ChIP-Qpcr validation of gain and loss H3K27ac enriched loci. (B-C) Motif analysis of gain and loss enrichment loci of H3K27ac in HFDHFB and HFDLFB group.

Fig S8

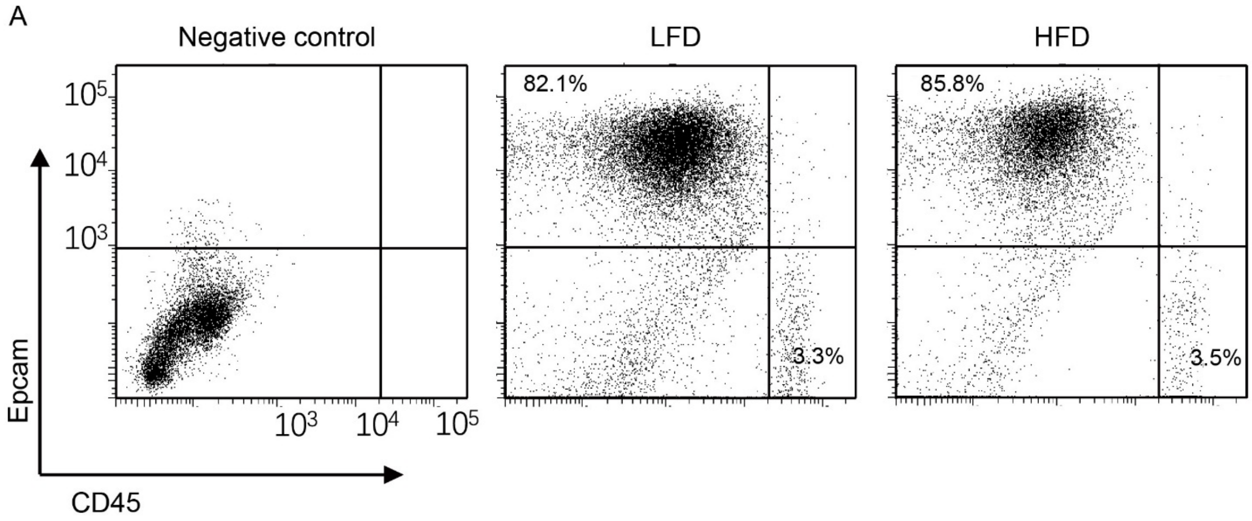

FigS8 Purity of isolated epithelial cells

(A) Fluorescence-activated cell sorting of pooled IECs labeled with antibodies marking either epithelial cells (EpCAM) or immune cells (CD45) reveal that [illegible]% of cells were epithelial (EpCAM positive and CD45 negative).
